# Arthroscopically Assisted Coracoclavicular Fixation Using a Single Flip Button Device Technique: What Are the Main Factors Affecting the Maintenance of Reduction?

**DOI:** 10.1155/2017/4859262

**Published:** 2017-08-02

**Authors:** Yong-Beom Lee, Jeehyoung Kim, Ho-Won Lee, Byung-Su Kim, Won-Yong Yoon, Yon-Sik Yoo

**Affiliations:** ^1^Department of Orthopedic Surgery, Hallym University Sacred Heart Hospital, Medical College of Hallym University, Anyang, Republic of Korea; ^2^Department of Orthopedic Surgery, Seoul Sacred Heart General Hospital, Seoul, Republic of Korea; ^3^Department of Orthopedic Surgery, Hallym University Dongtan Sacred Heart Hospital, Medical College of Hallym University, Dongtan, Republic of Korea

## Abstract

**Background:**

Among coracoclavicular (CC) fixation techniques, the use of flip button device was demonstrated to have successful outcomes with the advantage of being able to accommodate an arthroscopic procedure.

**Purpose:**

This study was conducted to investigate the factors associated with loss of fixation after arthroscopically assisted CC fixation using a single flip button device for acromioclavicular (AC) joint dislocations.

**Materials and Methods:**

We enrolled a total of 47 patients (35 men and 12 women). Plain radiography was performed at a mean of 24 months postoperatively to evaluate the final radiological outcome. The primary outcome measure was a long-term reduction of the AC joint for at least 24 months.

**Results:**

We found that 29 patients had a high quality reduction (61.7%) and 18 patients had a low quality reduction (38.3%) in initial postoperative CT findings. Our study showed that the duration (5 days) from injury to treatment and the quality of initial postoperative reduction were significantly associated with the maintenance of reduction at final follow-up.

**Conclusion:**

Our study showed that maintaining stable reduction after arthroscopically assisted CC fixation using a single flip button device technique is difficult especially in patients who received delayed treatment or whose initial reduction quality was poor.

## 1. Introduction

Acromioclavicular (AC) joint dislocations are commonly sustained by athletes, especially during contact sports or after a fall while skiing or cycling [1]. Various treatment methods for AC joint injury exist ranging from conservative treatment of asymptomatic low-grade dislocation to complex surgical reconstruction. Many surgical techniques that stabilize AC joint injuries have been described [2–8]. In particular, surgical techniques for coracoclavicular (CC) ligament fixation have been widely used in treating acute, high-grade AC dislocations. Among the recently developed CC fixation techniques, the flip button device has demonstrated successful outcomes with the advantage of being able to accommodate an arthroscopic procedure. However, there are concerns regarding complications related to metal subsidence with loss of CC reduction and risk of fractures in the coracoid process or in the clavicle despite the strong fixation strength, the minimal invasiveness, and the decreased morbidity with which this technique is associated. Further, earlier studies on Tightrope (Arthrex, Naples, FL, USA) fixation of AC dislocations by Defoort and Verborgt and by Thiel et al. have reported that fixation in one-third of patients with grade III and grade V failed [9, 10]. Surprisingly, Shin and Kim also demonstrated a high failure rate of over 50% associated with the use of single flip button device at the 2-year follow-up [11].

To the best of our knowledge, there are no studies that present guidelines to minimize short-term or long-term complications related to the use of flip buttons for AC joint dislocations. To this end, this study investigated the factors associated with fixation loss or reduction loss after arthroscopically assisted CC fixation using a single flip button device for AC dislocations. We hypothesized that a failure to attain long-term fixation may be associated with the initial reduction quality of the AC joint, which could be influenced by demographic, clinical, and radiological variables. Moreover, we investigated the effect of tunnel positions on the quality of AC joint reduction by standardizing the various tunneling points on the clavicle and the coracoid process to determine whether the initial quality of reduction is positively correlated with postoperative radiological outcomes.

## 2. Materials and Methods

### 2.1. Study Group Design

The study was a retrospective cohort study of prospectively collected data. The data was gathered from patients with AC dislocations who had been treated with arthroscopically assisted CC fixation using a single flip button device. The primary outcome measure was a long-term reduction of the AC joint for at least 24 months. The study was approved by our college's institutional review board, and all the patients provided informed consent before participation.

We enrolled a total of 47 patients (35 men and 12 women) who underwent surgery between January 2011 and February 2013. The inclusion criteria for the study were as follows: (1) patients who underwent arthroscopic AC joint reduction by a single surgeon (YYS) for an acute traumatic AC joint dislocation and (2) patients who received repairs using a single metal flip button (Arthrex Corp., USA). The exclusion criteria were as follows: (1) use of flip button combined with graft fixation for chronic injuries, (2) use of double flip buttons, (3) delayed surgical procedure (more than a month from the time of trauma), and (4) patients receiving a revision reconstruction of the AC joint as determined by plain AP radiography.

### 2.2. Data Collection

#### 2.2.1. Demographic Data

For the clinical variables, we collected data for age, gender, time of surgery, and the type of injury. The type of AC joint injury in these patients was classified according to the Rockwood classification [12]. Patient characteristics were stratified for diagnosis and are summarized in Table 1.

#### 2.2.2. Radiographic Data

*(1) Reduction Accuracy*. To evaluate immediate postoperative reduction, we used plain radiography and 3D CT. Plain radiography was also performed on average of 24 months postoperatively to evaluate the final radiological outcome.

We used the CC interval as our indicator of the initial and final reduction by measuring the vertical distance between the superior border of the coracoid and the inferior edge of the clavicle on standard anteroposterior radiographs. We rated final reduction on a dichotomous scale as either “good” or “poor.” A final CC distance of less than 2 mm difference in relation to that of the immediate postoperative CC distance was graded as good, and a final CC distance of more than 2 mm difference was graded as poor, which indicates a loss in reduction.

The quality of the immediate postoperative reduction was determined using 3D CT. We imaged all patients with a high-resolution 3D CT scanner (SOMATOM Sensation; Siemens, Erlangen, Germany) in 0.6 mm slices. Digital Imaging and Communications in Medicine files were obtained and imported into visualization software (Amira R 4.0; Mercury Computer Systems, Chelmsford, MA, USA) to construct virtual 3D models of the scapula and the clavicle.

**Figure 1:**
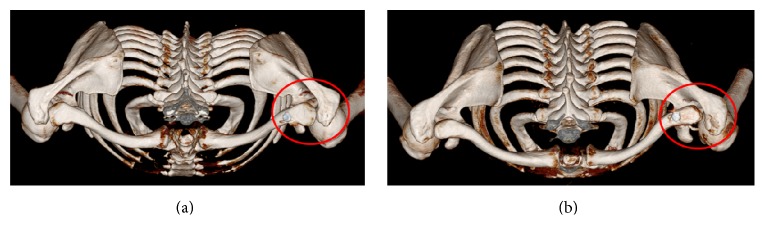
Relationship between distal clavicle and medial acromion with top view of 3D CT. (a) Location of distal clavicle with respect to medial acromion was almost identical to that of contralateral side of distal clavicle, indicating high quality reduction with less than 2 mm difference. (b) Distal clavicle was positioned posteriorly from corresponding articulation of medial acromion, indicating low quality reduction.

We defined a reduction to be of high quality when the distal clavicle was located perfectly with respect to the medial acromion or with less than a 2 mm difference of the contralateral AC joint on 3D CT scans (Figure 1(a)). Conversely, we defined a reduction to be of low quality when the clavicle showed a greater than 2 mm anterior or posterior displacement with respect to the contralateral AC joint on 3D scans (Figure 1(b)).

**Figure 2:**
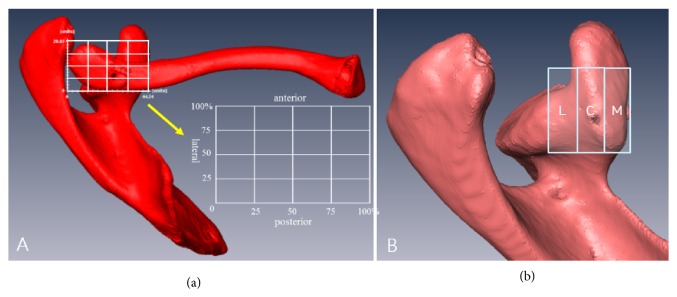
Virtual 3D models of the clavicle and scapular complex were created in a top (superior) view. (a) Grid system of distal clavicle was defined as the view with the widest superior surface of clavicle. (b) The grid encompassing the coracoid process was divided into three blocks along the lateral to medial axis (L: lateral block, C: central block, and M: medial block).

*(2) Tunnel Accuracy*. All virtual 3D models of the clavicle and scapular complex were created in a top (superior) view. A top view was defined as the view with the widest superior surface of clavicle, with the posterior surface of the distal clavicle that is parallel to the horizontal axis and with the coracoid process facing as anteriorly as possible (Figure 2(a)).

Clavicle and coracoid tunnel distributions were evaluated using a customized grid system described by Bernard et al. [13]. The anterior margin of the grid was set at the most anterior part of distal clavicle, the posterior margin at the posterior cortex of distal clavicle, the medial margin at the medial surface of the coracoid process, and the lateral margin at the most lateral tip of the clavicle (Figure 2(a)). The grid encompassing the distal clavicle was divided into four large blocks along the lateral to medial (*x*) and the posterior to anterior (*y*) axis, and each block was further divided into five small blocks. The grid encompassing the coracoid process was divided into three blocks along the lateral to medial axis. The medial block contained the medial half of the coracoid process. The central block represents the lateral half of the coracoid process. The lateral block was set in outside of coracoid process towards the glenoid neck (Figure 2(b)). The widths of all blocks were equal. Each tunnel location was mapped onto the grid system as coordinates. Two independent examiners examined the tunnel placement on all postoperative CT scans and reviewed the quality of the AC reduction. Repeated measurements were performed at a minimum of 3-week intervals to calculate intrarater reliability.

### 2.3. Statistical Analysis

Standard descriptive statistics were used to analyze patient characteristics. Continuous variables were summarized into either mean and standard deviation (SD) or median and interquartile range (IQR), according to distribution. Dichotomous variables were summarized as percentages. The study population was divided into 2 categories (reduction loss group or reduction maintenance group) based on a radiological evaluation at the final follow-up. Successful reduction at final follow-up was defined as a final CC distance of less than 2 mm difference in relation to that of the immediate postoperative CC distance measured on standard anteroposterior radiographs. We measured the dependent variable binomially (reduction or no reduction) based on the radiological outcome. We analyzed the resultant data through logistic regression and tree analysis. Each independent variable and its respective dependent variables were analyzed using the logistic regression model. Because we hypothesized that distal clavicular tunnel position is important for reduction, we performed an additional logistic regression analysis to evaluate its effect as coordinates along *x*-axis and *y*-axis on the quality of reduction. The second analysis consisted of generating a decision tree for the overall group. We represented the node splitting of the data into separate groups as circles (Figure 4).

We set statistical significance at *P* values < .05. A multiplicity was not taken into account during our analysis. We used dBSTAT (ver. 5.1, dBSTAT Software, Seoul, Korea) for logistic regression and R (version 3.2.3 party package) for generating a regression tree. All graphical illustrations were created in Excel (Microsoft® Excel 365).

## 3. Results

**Figure 3:**
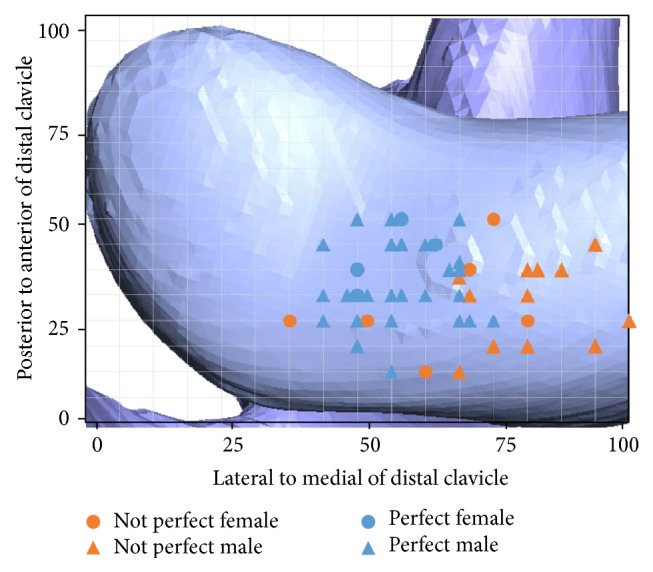
3D standardization model of distal clavicle displaying tunnel locations with coordinates. “Perfect” means high quality reduction.

The mean age of the patients was 37 years (range: 20–54 years). The CC distance of all patients returned to normal on postoperative plain X-rays. Among them, we found that 29 patients had a high quality reduction (61.7%) and 18 patients had a low quality reduction (38.3%) in terms of initial postoperative CT findings (Figure 3).

Radiological examination at the time of final follow-up revealed that reduction was maintained in 31 patients, of whom 24 had had an initial high quality reduction. Our findings showed that the type of injury classified according to the Rockwood classification was not associated with the status of reduction at the final follow-up (*P* = .42).

Using univariate logistic regression, we assessed the factors influencing reduction in patients at final follow-up radiography. The results of our analysis significantly differed between those who had treatment within 5 days of injury and those who had treatment after 5 days (*P* = .020; OR = 1.35). Interestingly, our data also showed that successful initial reduction was carried through to the final follow-up significantly (*P* = .018; OR = 4.79). Further, our findings suggested that having an eccentric tunnel location in the clavicle was extremely rare in patients with high quality reduction. We also found that the majority of clavicle tunnels with high quality reduction were located, with statistical significance, at the midline, along the lateral to medial (*x*) axis of the grid (*P* < .05).

However, the position of the coracoid process tunnel had no statistical correlation with the outcome of reduction at the final follow-up. Gender also did not affect long-term reduction (Table 2).

We were able to attain similar results for univariate and multivariate regression analysis. As in the univariate analysis, the multivariate analysis showed that the duration from injury to treatment (*P* = .002; OR = 1.82) and the quality of initial postoperative reduction (*P* = .003; OR = 59.71) were significantly associated with the maintenance of reduction at final follow-up (Table 3).

**Figure 4:**
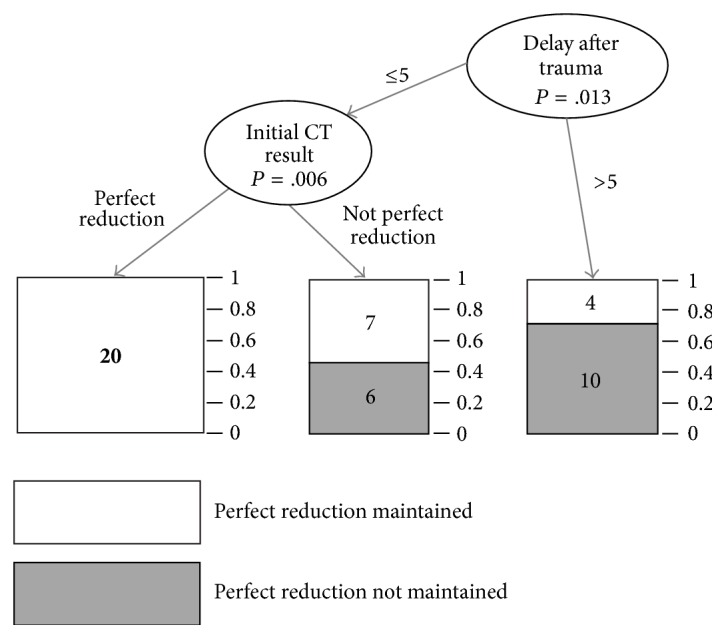
Tree analysis of reduction maintenance. Delay after trauma means duration from injury to treatment, and initial CT result means initial reduction quality.

As an additional statistical analysis, we employed classification tree analysis, where both duration from injury to treatment (*P* = .013) and initial reduction quality (*P* = .006) were found to be statistically significant factors. Of the patients who underwent surgery within 5 days after trauma, there were 27 patients whose reduction was maintained until the last follow-up and 6 patients were not maintained. Of the patients who underwent surgery after 5 days, there were only 4 patients whose reduction was maintained until the last follow-up and 10 patients were not maintained. Of the patients who underwent surgery within 5 days after trauma, all 20 patients who had good initial reduction quality were well maintained in reduction. Of the patients who had poor initial reduction quality, there were 7 patients whose reduction was maintained until the last follow-up, but 10 patients were not maintained (Figure 4). Our results had a sensitivity of 0.87 and a specificity of 0.62.

## 4. Discussion

We found that achieving long-term reduction after arthroscopically assisted CC fixation using a single flip button device technique is difficult when treatments are delayed or initial reduction quality is poor. Our multivariate logistic regression analysis revealed that the duration from trauma to treatment (*P* = .002; OR = 1.82) was the most important factor influencing reduction at the final follow-up. Among patients with favorable initial reduction, we were able to conclude from our findings that those with more lateralized clavicle tunnels had better final reduction than those with less lateralized clavicle tunnels. Tunnel position of the coronoid process, conversely, did not affect reduction.

Our study also showed that the proportion of failed reduction increased as the clavicle tunnel was more medially, implying that a medial position of clavicle tunnels was more likely to lead to a poor reduction outcome. This may be because the tunnel direction becomes diagonal rather than vertical relative to the outer coracoid process anatomically when clavicle tunnels are placed near the conoid process, which leads to ineffective scapuloclavicular biomechanics. Then, not only the initial quality of the reduction but also the long-term reduction is compromised.

In fact, Rios et al. [14] proposed that the ratio of the distance to the medial border of the conoid divided by the clavicle length is on average 0.3. As noted by them, biomechanical studies have shown that the conoid tunnel is the most important factor resisting translation beyond the trapezoid [15, 16]. Cook et al. [17] reported that the medialization of the conoid bone tunnel leads to a significantly high rate of unsuccessful reduction after reconstruction. They reported that patients with a medial tunnel ratio of ≥0.3 had failed results but all patients with a conoid tunnel ratio of <0.25 did not fail. Other studies have reported that 28.6% of patients could not maintain intraoperative reduction, and they also found that more medial bone tunnels were found in them than those with successful reconstructions [18–20].

The main goal of this procedure is to approximate the stubs of the torn CC ligaments and preserve AC joint reduction until the ligaments have healed. Advantages of this procedure include minimal invasiveness, better visualization of the coracoid for optimal fixation, less damage to the surrounding soft tissue, and, therefore, less interference to the primary ligament healing. In particular, arthroscopically assisted flip button tech allows a nonrigid fixation of the AC joint allowing a more anatomic reconstruction than other widely used techniques such as CC slings, hook plates, or the Weaver-Dunn procedure. Despite this, there are concerns regarding how well this method can provide horizontal stability and the complications related to metal subsidence with gradual loss of reduction. Although the two-endobutton technique has been described to overcome these concerns, it is still a challenge for clinicians to place two holes in the coracoid process and clavicle without risking fracture of either bony structure.

However, when a proper single tunnel is holed in place, it allows for fine horizontal rotation of the scapula and clavicle which normally occurs. By creating only one tunnel, it is possible to minimize the additional damage to an already damaged CC ligament during tunnel formation, as well as anatomically aligning the acromion and clavicle through fine horizontal rotation of the scapula and clavicle. Thus, this single tunnel approach can theoretically improve initial reduction quality of AC joint reconstruction and reduces the loss of reduction at the final follow-up. In this context, understanding the clustering results of the clavicle tunnels is important for long-term reduction as well as for initial reduction quality.

As shown in Figure 3, the clavicle tunnels that achieved initial high quality reduction were distributed to the midline along the transverse axis of the clavicle and laterally to the longitudinal axis of the coracoid process. However, the bone mineral density tends to decrease towards the lateral end of the clavicle compared to the medial side, and reports have shown that bone mineral density in the region of 20–50 mm of the lateral edge has the most optimal density [21]. Thus physicians should know the possibility of reduction failure to patients with low bone mineral density if clavicle tunnel position is too much lateralized. Our study showed that duration between injury and treatment is another determinant of long-term reduction at the final follow-up. Specifically, our subanalysis (logistic regression and tree analysis) showed that patients who had received treatment within 5 days of the injury had a statistically lower rate of reduction failures at the final follow-up than those who had received treatment after this period. Therefore, for patients undergoing surgical intervention, an early diagnosis and expedited decision for surgery may reduce chances of failed radiological reduction. If patients with delayed treatment undergo treatment using single tunnel flip button technique, they should be advised of the difficulties in attaining a stable reduction, as well as of other adjunct or alternative treatments such as ligament reconstruction, prior to treatment.

**Figure 5:**
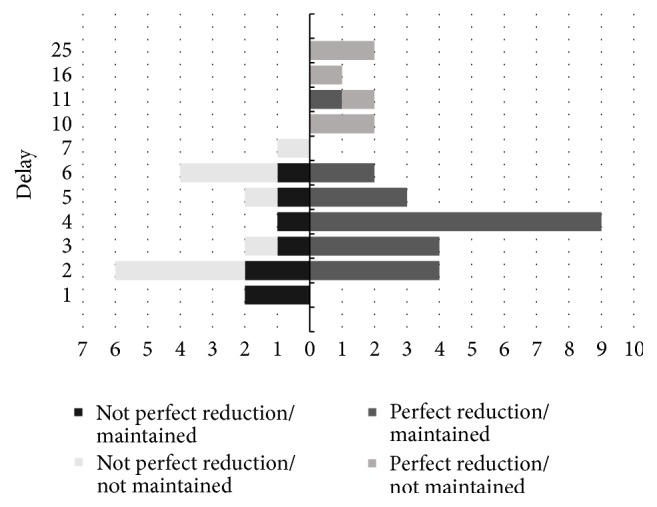
Relationship between the quality of initial reduction and the reduction at final follow-up. Vertical axis (delay) is duration from injury to operative treatment. Horizontal axis is the number of each patient.

Further, our results showed that a vast majority of patients who received treatment within 7–10 days of injury and had a successful initial reduction had failed to show reduction by the final follow-up (6 of 7 patients) (Figure 5).

We can hypothesize that with the delay in treatment the resorption of hematoma had progressed and the soft tissue had stabilized leading to better initial reduction than we would have anticipated with treatments performed earlier. Yet in spite of the promising initial reduction the long-term reduction shows a high failure rate in these subsets of patients; thus, for patients who are to receive treatment after 7–10 days of the injury, we recommend that stronger fixation, using adjunct fixation devices or open reduction, as opposed to an anatomical reduction be prioritized.

This study had several limitations. First, although we had acquired our data prospectively (through radiography and postoperative follow-up examinations), the study design itself was retrospective in nature and the time of final follow-up was inconsistent between patients. Second, our study population was not large enough to determine the reliability of our findings; specifically, the reliability of our overall rate of high quality reduction (57.4%) was not assessed. Third, individual anatomic variations were not fully taken into consideration. Because we evaluated tunnel position through reconstructed images, potential nonuniformed repositioning of 3D virtual models may have led to misinterpretation of the best fit tunnel position. Fourth, because only static horizontal stability and not dynamic horizontal instability was reliable, the effect of persistent or recurrent dynamic horizontal instability on the clinical follow-up remains unclear.

Our study has many strength points notwithstanding these limitations. We used minimally invasive arthroscopic reconstruction with a single clavicle tunnel. Next, our study proposed two novel predictors of surgery outcomes—duration from injury to treatment and the initial reduction quality. Further, we attempted to make an accurate assessment of the reduction by using 3D computed tomography to generate 3D views of the reconstruction site as opposed to 2D views. We used statistical tools such as logistic regression analysis and tree analysis to identify groups of values that occur along a continuum as continuous variables and by doing so avoid the possibility of dividing a potential group and maximize our ability to identify important groups. Lastly, we found that our study had a sensitivity of 0.87 and a specificity of 0.62, which demonstrates a high clinical sensitivity. In all, we believe that the findings of our study will guide physicians in decision-making and in predicting the prognosis of patients after AC-CC reconstruction.

## 5. Conclusion

To sum up, we found that maintaining stable reduction after arthroscopically assisted CC fixation using a single flip button device technique is difficult especially in patients who received delayed treatment or whose initial reduction quality was poor (due to a medially positioned clavicle tunnel). We anticipate that our results will aid in the treatment and the prediction of prognosis in patients with AC-CC joint injury.

## Figures and Tables

**Table 1:** Patient demographics.

Characteristics	Value
Age, yrs, mean (range)	37 (20–54)
Sex, *n*	
Male	35
Female	12
Injury type, *n*	
Direct	41
Indirect	6
Timing of surgery, *n*	
Acute	40
Chronic	7
Quality of reduction, *n* (%)	
High	29 (61.7)
Low	18 (38.3)
Rockwood classification, *n* (high : low)	
Type III	15 (11 : 4)
IV	2 (0 : 2)
V	30 (18 : 12)

**Table 2:** Comparison of reduction maintenance in univariate logistic regression analysis^a^.

	*P* value	OR	95% CI
Sex	.720	1.32	0.31–6.93
Clavicle tunnel position			
*x*-axis on grid	.094	1.27	0.97–1.71
*y*-axis on grid	.702	0.93	0.64–1.34
Coracoid tunnel position			
Central	.992		
Lateral	.993		
Medial	.992		
Duration (within 5 days)	**.020**	1.35	1.10–1.82
Initial reduction (Good)	**.018**	4.79	1.36–18.54

^a^Bolded *P* values indicate a statistically significant difference (*P* < .05). OR: odds ratio.

**Table 3:** Comparison of reduction maintenance in multivariate logistic regression analysis^a^.

	*P* value	OR	95% CI
Clavicle tunnel position (*x*-axis)	.454	1.19	0.77–2.00
Duration (within 5 days)	**.002**	1.82	1.32–2.87
Initial reduction (Good)	**.003**	59.71	6.71–1855.80

^a^Bolded *P* values indicate a statistically significant difference (*P* < .05). OR: odds ratio.

## Additional Points

*What Is Known about the Subject. *Arthroscopic CC fixation techniques with a single flip button have concerned about a gradual loss of reduction over time due to complications related to metal subsidence or tunnel widening in both clavicle and coracoid.* What This Study Adds to Existing Knowledge*. This study presents guidelines to minimize long-term complications related to the use of single flip button for AC joint dislocations.
